# Spontaneous Pneumomediastinum and Diffuse Subcutaneous Emphysema after Methamphetamine Inhalation

**DOI:** 10.1155/2020/7538748

**Published:** 2020-03-07

**Authors:** Michael Agustin, Gabriel David, Ji Yeong Kang, Ornusa Teerasukjinda

**Affiliations:** Guam Regional Medical City, 133 Route 3, Dededo, Guam, USA 96929

## Abstract

Methamphetamines are commonly abused drugs for their stimulant and euphoric effects. Inhaled and intravenous use may cause damage to the respiratory system. Spontaneous pneumomediastinum is a condition where changes in intrathoracic pressure leads to alveolar rupture and dissection of air along the tracheobronchial tree. Massive subcutaneous emphysema may result from pneumomediastinum which may compromise the central airway. In this case report, we present an unusual case of spontaneous pneumomediastinum and severe subcutaneous emphysema following inhalation of methamphetamine. This case emphasizes the rising concern on the acute respiratory complications of methamphetamine use.

## 1. Introduction

Inhaled and intravenous use of stimulants may cause a variety of respiratory injuries. These injuries include but are not limited to nasal septum perforation, pulmonary vascular disease, pulmonary hemorrhage, thermal injury, pulmonary edema, and pneumothorax [1]. Spontaneous pneumomediastinum is a benign condition usually caused by alveolar rupture with subsequent air tracking along the tracheobronchial tree. Emesis and asthma flare-ups may trigger free air in the mediastinum; however, in many cases, there is no triggering event identified [2]. Illicit stimulants such as cocaine, methamphetamine, ecstasy, and mephedrone have been associated with pneumomediastinum [3–7]. Subcutaneous emphysema may occur when air from the ruptured bronchoalveolar wall escapes into the subcutaneous plane. Massive subcutaneous emphysema may lead to acute airway compromise [8].

## 2. Case

**Figure 1:**
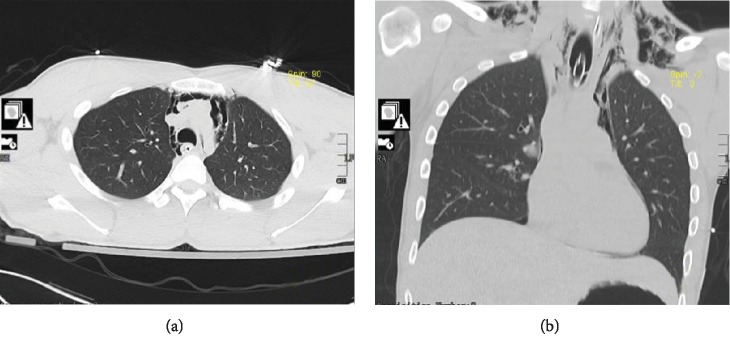
(a) Chest computed tomography (CT) axial view showing extensive pneumomediastinum with air dissecting to the peribronchovascular interstitial sheaths and visceral pleura. (b) Chest computed tomography (CT) coronal view showing extension of the subcutaneous emphysema on the supraclavicular area.

**Figure 2:**
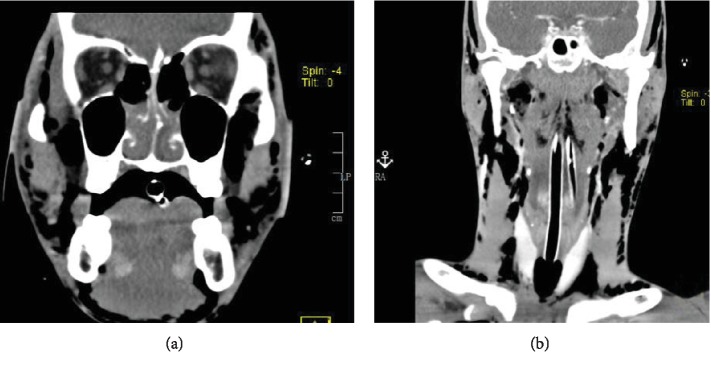
(a) Head computed tomography (CT) coronal view showing extension of subcutaneous emphysema into the orbits and superficial bitemporal soft tissues. (b) Neck computed tomography (CT) coronal view showing massive soft tissue emphysema enveloping the strap muscles of the upper chest, neck, and deep facial spaces of the neck.

A twenty-two-year-old previously healthy male presented to the emergency department for worsening facial and neck swelling. He noticed worsening dyspnea accompanied by facial swelling a few hours after smoking methamphetamine. Within a few minutes in the emergency room, the patient's mental status declined with more labored breathing; thus, the patient was electively intubated for airway protection. Differential diagnosis was acute allergic reaction to illicit drugs causing upper airway compromise versus acute inhalational injury. Initial chest computed tomography (CT) showed extensive pneumomediastinum with air dissecting to the peribronchovascular interstitial sheaths, interlobular septa, and visceral pleura (Figures 1(a) and 1(b)). There was no evidence of pneumothorax. CT of the neck also showed massive soft tissue emphysema enveloping the strap muscles of the upper chest, neck, deep facial spaces of the neck with extension into the orbits, axillary compartment, and superficial bitemporal soft tissues (Figures 2(a) and 2(b)). Urine toxicology screening confirms methamphetamine use. The patient also had elevated creatinine kinase, elevated lactate level, and leukocytosis.

Mechanical ventilation was adjusted having very minimal to no positive expiratory pressure (PEEP) and prolonged expiratory phase. Placement of chest blow holes was not pursued as swelling on the face and neck improved after a few hours. The suspicion for tracheobronchial tree injury was low; thus, we did not perform bronchoscopic surveillance of the airway. Patient received empiric antibiotic treatment with ampicillin sulbactam. The patient was extubated in less than 24 hours after the improvement of peak inspiratory and plateau pressures with the positive cuff leak test. Repeat chest imaging showed decreased subcutaneous emphysema and resolution of pneumomediastinum. No dietary restriction was recommended after extubation as there was no evidence of aerodigestive organ injury. The patient was discharged after 72 hours with improved symptoms.

## 3. Discussion

Pulmonary complications have been reported with the use of illicit stimulants. Some recognized respiratory complications are nasal septum perforation, pulmonary vascular disease, pulmonary hemorrhage, pulmonary edema, and pneumothorax [1]. Our case focuses on the uncommon complication of methamphetamine use in which spontaneous pneumomediastinum led to severe subcutaneous emphysema and central airway compromise. Spontaneous pneumomediastinum is the presence of free air in the mediastinum without any obvious primary pathologic event such as trauma, intrathoracic infections, or violation of the aerodigestive track [2]. It is postulated that a sudden increase in intrathoracic pressure results in increased intra-alveolar pressure. The pressure difference leads to alveolar rupture with further leakage of air throughout the interstitium and bronchovascular tissue sheath. This air leak follows a centripetal pattern toward the mediastinum and may extend to the fascial planes of the neck, as the air follows the path of least resistance. Forced Valsalva maneuvers are usually practiced in the use of illicit stimulants to enhance drug absorption. This forced Valsalva creates an intrathoracic pressure differential causing alveolar rupture. This maneuver is mostly reported with cocaine where users commonly inhale deeply and then perform a ritualistic Valsalva maneuver to accentuate the absorption and effects of the drug [9, 10]. Another proposed possibility of alveolar rupture in illicit drug users was the presence of contaminants in the drug preparations causing alveolar injury [2]. There are limited case reports on pneumomediastinum with concomitant use of methamphetamine, ecstasy, and mephedrone [3–5, 7]. In this particular case, pneumomediastinum occurred due to inhalational injury from methamphetamine. However, the central airway compromise from subcutaneous emphysema triggered more forceful breathing and rapid increase in intrathoracic pressure causing a cascade of worsening subcutaneous emphysema.

Plain chest X-ray including computed tomography (CT) is an adequate evaluation procedure after a thorough history and physical examination to exclude secondary pneumomediastinum [11]. In one case series, only 69% of the cases were discovered by chest radiograph, with the remaining 31% found on chest CT scan [2]. In patients with secondary pneumomediastinum, plain chest X-ray has lower sensitivity for the diagnosis [2]. Overall, computed tomography (CT) has become the gold standard for diagnosing pneumomediastinum. When aerodigestive organ injury is considered, further invasive interventional studies such as bronchoscopy or esophagogram should be performed.

Spontaneous pneumomediastinum is often a self-limiting disease; however, due to concerns for the integrity of the aerodigestive tract, this finding usually results to unnecessary radiological investigations and antibiotic use. In one case series, spontaneous pneumomediastinum is overinvestigated and overtreated; thus, clinicians need to be more judicious with the use of hospital resources in managing patients with spontaneous pneumomediastinum [12]. For this particular case, mechanical ventilation was necessary due to rapid central airway compromise. Having a patent airway with intubation decreased intrathoracic pressure and led to overall improvement of pneumomediastinum and subcutaneous emphysema. We have provided very minimal to no PEEP so as not to aggravate elevated alveolar pressure. Overall, rest and pain control are the main components of conservative management. Oxygen therapy has been recommended as the consumption of oxygen increases the diffusion pressure of nitrogen in the interstitium, which promotes absorption of mediastinal free air. The role of empiric antibiotic treatment remains controversial, and is mostly recommended in patients with leukocytosis and fever [11]. In the absence of suspected aerodigestive injury, dietary restriction is not warranted. Reported rates of recurrence are very low and routine follow-up is seldom recommended. In conclusion, spontaneous pneumomediastinum is an uncommon complication of methamphetamine use and life-threatening sequela may ensue with massive subcutaneous emphysema compromising patency of the central airway. Prompt identification is needed to recognize this complication to prevent morbidity or morality. This case adds increasing concern on the respiratory complication of methamphetamine use.
